# First tracks of newborn straight-tusked elephants (*Palaeoloxodon antiquus*)

**DOI:** 10.1038/s41598-021-96754-1

**Published:** 2021-09-16

**Authors:** Carlos Neto de Carvalho, Zain Belaústegui, Antonio Toscano, Fernando Muñiz, João Belo, Jose María Galán, Paula Gómez, Luis M. Cáceres, Joaquín Rodríguez-Vidal, Pedro Proença Cunha, Mario Cachão, Francisco Ruiz, Samuel Ramirez-Cruzado, Francisco Giles-Guzmán, Geraldine Finlayson, Stewart Finlayson, Clive Finlayson

**Affiliations:** 1 Naturtejo UNESCO Global Geopark, Geology Office of the Municipality of Idanha-a-Nova, Idanha-a-Nova, Portugal; 2 Instituto D. Luiz, University of Lisbon, Lisbon, Portugal; 3 Departament de Dinàmica de la Terra i de L’Oceà, Facultat de Ciències de la Terra, Institut de Recerca de la Biodiversitat (IRBio), Universitat de Barcelona (UB), Barcelona, Spain; 4 Departamento de Ciencias de la Tierra, Universidad de Huelva, Huelva, Spain; 5 Departamento de Cristalografía, Mineralogía y Química Agrícola, Universidad de Sevilla, Seville, Spain; 6 Geosciences Center, University of Coimbra, FlyGIS-UAV Surveys, Coimbra, Portugal; 7 Centro Administrativo del Acebuche, Parque Nacional de Doñana, Matalascañas, Huelva, Spain; 8 Department of Earth Sciences, MARE-Marine and Environmental Sciences Centre, University of Coimbra, Coimbra, Portugal; 9 Department of Geology, Faculty of Sciences, University of Lisbon, 1749-016 Lisbon, Portugal; 10 The Gibraltar National Museum, Gibraltar, UK; 11 Institute of Life and Earth Sciences, University of Gibraltar, Gibraltar, UK; 12 Department of Life Sciences, Liverpool John Moores University, Liverpool, UK; 13 Department of Anthropology, University of Toronto, Scarborough Campus, Toronto, Canada

**Keywords:** Palaeontology, Palaeoecology

## Abstract

Tracks and trackways of newborns, calves and juveniles attributed to straight-tusked elephants were found in the MIS 5 site (Upper Pleistocene) known as the Matalascañas Trampled Surface (MTS) at Huelva, SW Spain. Evidence of a snapshot of social behaviour, especially parental care, can be determined from the concentration of elephant tracks and trackways, and especially from apparently contemporaneous converging trackways, of small juvenile and larger, presumably young adult female tracks. The size frequency of the tracks enabled us to infer body mass and age distribution of the animals that crossed the MTS. Comparisons of the MTS demographic frequency with the morphology of the fore- and hind limbs of extant and fossil proboscideans shed light into the reproductive ecology of the straight-tusked elephant, *Palaeloxodon antiquus*. The interdune pond habitat appeared to have been an important water and food resource for matriarchal herds of straight-tusked elephants and likely functioned as a reproductive habitat, with only the rare presence of adult and older males in the MTS. The preservation of this track record in across a paleosol surface, although heavily trampled by different animals, including Neanderthals, over a short time frame, permitted an exceptional view into short-term intraspecific trophic interactions occurring in the Last Interglacial coastal habitat. Therefore, it is hypothesized that Neanderthals visited MTS for hunting or scavenging on weakened or dead elephants, and more likely calves.

## Introduction

The straight-tusked elephant *Palaeoloxodon antiquus* Falconer & Cautley is among the most powerful proboscideans that has ever lived, and one of the most robust of the Elephantinae species, with very wide heads carrying extremely long tusks. Based on well-preserved skeletons, estimates of maximum shoulder height varies from 300 to 420 cm and body mass from 4.5–5.5 to 13 tonnes for females and males, respectively^1^. Recent genetic analyses place *Palaeoloxodon antiquus* as closely related to the more modest African forest elephant *Loxodonta cyclotis* Matschie^2^. According to Palkoupoulou^3^, straight-tusked elephants descended from a mixture of three ancestral populations related to the African elephant *Loxodonta africana* Blumenbach, the woolly mammoth *Mammuthus primigenius* Blumenbach and the present-day African forest elephant *L*. *cyclotis*. *Palaeoloxodon antiquus* dispersed rapidly in Europe around 1 million years ago^4,5^ replacing the southern mammoth *Mammuthus meridionalis* Nesti. *Palaeoloxodon antiquus*, *M*. *trongontheri* Pohlig and *M. primigenius* were the proboscideans coexisting in Europe between latest Early Pleistocene to about MIS 3; the steppe mammoth became extinct in Europe after Middle Pleistocene^4^. However, only very rarely they were found together in the same latitudes, the forest-browser *P. antiquus* being common in interglacial periods and the grazer mammoths typical of glacial steppes^4^. This is especially evident in the southernmost latitudes of Europe.

In the Iberian Peninsula, the straight-tusked elephant prevailed in Mediterranean evergreen woodland which was widespread during the interglacial phases, such as the Last Interglacial^5^. This is especially true in southern Spain, where *P. antiquus* replaced *M. trogontherii* during Middle Pleistocene^6^. In response to climate cooling after the MIS 5a, and the consequent dramatic vegetation changes throughout Europe, populations of *P. antiquus* were progressively reduced until left only in refugia, mainly but not exclusively in southern Europe^4,5,7^, including southernmost Iberia. On the other hand, the cold-adapted *Mammuthus primigenius* only occasionally extended its maximum geographical presence towards the south of the peninsula during the coldest periods of the Late Pleistocene^8^, reaching El Padul basin at Granada (Southern Spain), during the Last Glacial Period (MIS 3a). *Palaeoloxodon antiquus* had its last vestiges in the same period and the same biogeographical area^4,9–11^.

The functional morphology of their bones, their ancient DNA, the isotopic composition of their teeth, or even evidence of predation (including by hominin hunting) are fundamental data to understand the biology of elephants. However, for behavioural and ecological interpretations, the track fossil record provides substantial in situ and in vivo information usually not obtainable from the skeletal record^12^. Proboscidean-type tracks and trackways are found occasionally in the fossil record worldwide and they provide valuable and, sometimes, the only possible information about behaviour, locomotor characteristics and social structure of their trackmakers^13^.

**Figure 1:**
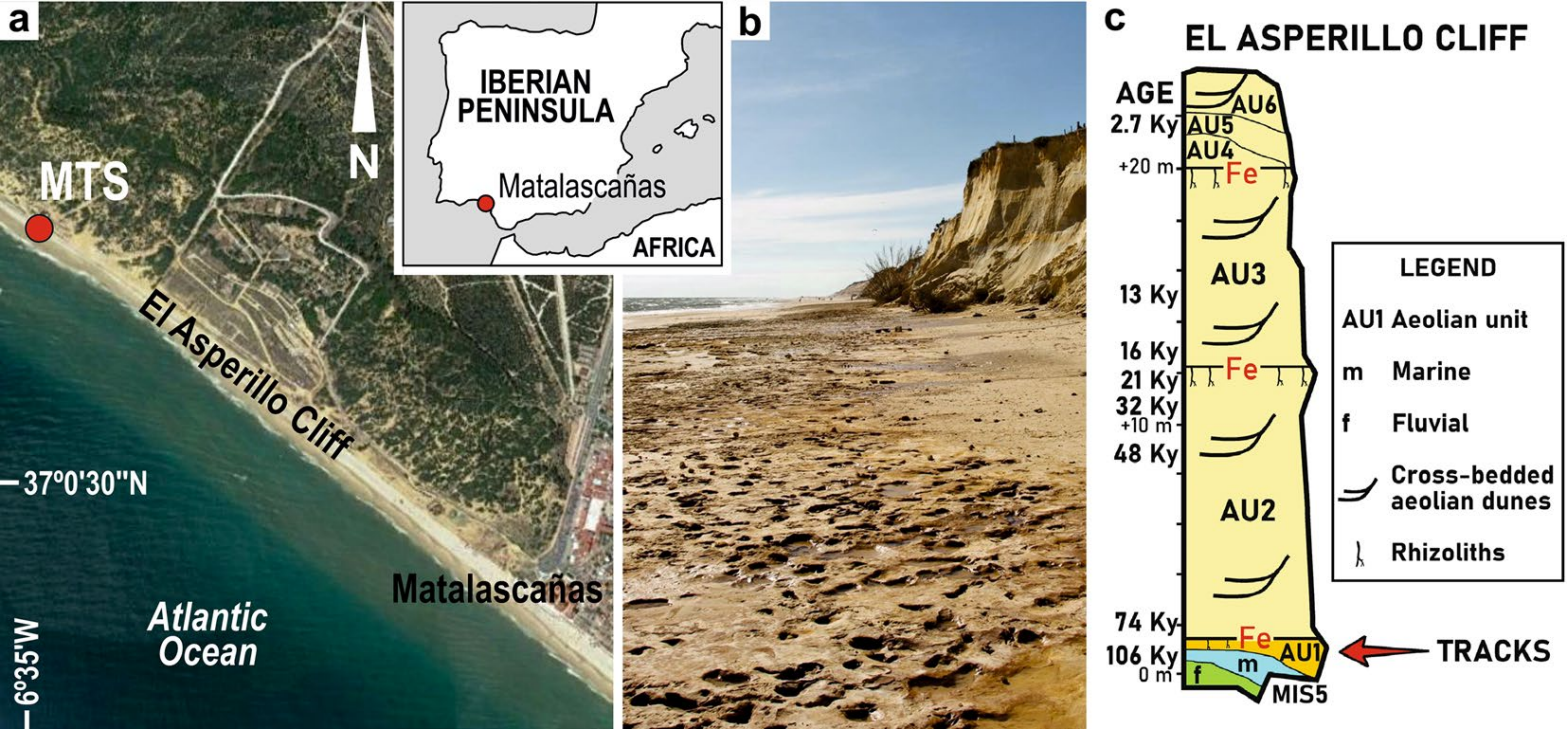
(**a**) Geographic setting of the study area and its location at the Iberian Peninsula. (**b**) General view of the ‘Matalascañas Trampled Surface’ ephemerally exposed during 2020 spring storm surges and usually covered by a thick blanket of beach sand. (**c**) Simplified stratigraphic section of the ‘El Asperillo’ Cliff^15^.

An Upper Pleistocene stratigraphic surface with vertebrate tracks and trackways, named the ‘Matalascañas Trampled Surface’ (MTS)^14^, has been exposed at the base of the ‘El Asperillo’ cliff (section 37°01′07″ N–6°35′17″ W to 37°00′36″ N–6°34′23″ W) near Torre de la Higuera at the municipality of Almonte, urban area of Matalascañas, in the vicinities of Doñana National Park (Matalascañas, Huelva, SW Spain). A significant part of MTS located in the mesotidal range was uncovered as a result of the 2020 spring storm surges (Fig. 1). The exposure was ephemeral since most of the time the coastal area is mantled by > 1.5 m beach sand (Fig. S1). The MTS has been identified as occurring at the base of the informal lithostratigraphic unit AU1, according to the local stratigraphic section, which was deposited during the MIS 5 (dated by OSL as 106 ± 19 ky^15^). Neto de Carvalho et al.^15,16^ pointed out that this trampled surface developed within the paleosol^17^, records an exceptional single event where tracks and trackways of different vertebrates ascribed to Artiodactyla (*Bos primigenius*?, *Cervus elaphus* and *Sus scrofa*), Elephantidae (*Palaeoloxodon antiquus*), Canidae (*Canis lupus*) and waterbirds (Anserinae and Charadrii) were preserved. Most recently, Mayoral et al.^18^ described Neanderthal footprints, some of them clearly located in the MTS. The MTS ichnoassociation is characterized by the ichnogenera *Cervipeda* Vialov/*Bifipides* Demathieu et al. and *Suidichnus galani* Neto de Carvalho et al. as artiodactyl traces, the ichnogenus *Probiscipeda* for *P. antiquus* traces, the ichnogenus *Hominipes* for Neanderthal footprints, the ichnogenus *Canipeda* Panin and Avram for wolf traces and, the bird traces could belong to the ichnogenus *Presbyorniformipes* Yang et al., in addition to other tetradactyl ichnogenera still not studied in detail^14,16^. The MTS is interpreted, from the sedimentological point of view, as a hydromorphic paleosol with abundant rhizoliths developed during the last interglacial period^17^, in a temporarily exposed interdunal area, similar to current examples in the Doñana National Park. Ferriargillan coating and the first eolian rippled sands of AU1 may have been responsible for the long-term preservation of the MTS traces from erosion^16^. However, ever present intertidal erosion upon poorly cemented fine- and clayey sandstones clearly affects the tracks and rhizoliths when exposed from the beach sand cover, blurring morphological features after few tides and deepening the trace fossils. This is clearly evident by the many undetermined weathered structures and potholes described as hominin footprints^18^.

Nevertheless, non-weathered exposures of MTS provide a unique sedimentary record of the coastal habitat with abundant and well-preserved proboscidean tracks and a few discernible trackways. This important finding raises the question about the ecological reasons for the presence of *Palaeoloxodon antiquus*, likely in herd, in the coastal environments of SW Spain during the beginning of the Late Pleistocene, as well as the coeval presence of Neanderthals. The MTS reveals new data on *Palaeoloxodon antiquus* locomotor behavior, social group structure and ecology based on age frequency estimates from tracks, and possible trophic interactions. Age estimation and population age structure of *P. antiquus* from footprint dimensions follows methodologies currently used to study elephant populations in Africa and Asia. In particular, the well-preserved small proboscidean footprints preserved in MTS show, for the first time, the presence of calves and juveniles, some younger than 2 months to 2 years of age, with important implications for understanding the reproductive ecology of *P. antiquus*.

### The ichnological record of proboscideans

The ichnological description of the proboscidean tracks and trackways present in the MTS can be found below, and in the supplementary Table S1 in comparison with the known trace fossil record. Proboscidean tracks are known from as early as the Late Eocene of Iran^19^ to the latest Pleistocene attributed to woolly mammoths^20,21^. They show for different groups of proboscideans rather similar morphological features, emphasizing the need for an ichnotaxonomic revision of the ichnogenus *Proboscipeda*. Irrespective of this, the size distribution of proboscidean tracks varies enormously, along the phylogeny, and even within the same population, when considering sexual dimorphism and ontogeny. The smallest footprint attributed to a woolly mammoth calf is 11 cm in length^22^ and the largest *P. antiquus*-related tracks may have reach over 70 cm ^11^. Lucas et al.^22^ proposed to apply the ichnogenus *Proboscipeda* Panin & Avram to all proboscidean fossil footprints. *Proboscipeda* is described as large oval to subcircular tracks, with large and flat sole surface either ornamented or smooth, three to five short and blunt toe impressions pointing anteriorly, and with deeper tracks impressing a noticeable marginal ridge (emended diagnosis by Aramayo et al.^23^). *Manus* impressions are more circular than the *pes*, which is common in proboscidean tracks and reflects subtle anatomic differences. The hindfoot is usually more plantigrade-like and the forefoot more digitigrade in functional posture, the fore limbs supporting around 60% of the body weight in extant elephants^24^. The number of toes may be indistinctly three to five, either in fore- and hindfeet, such as in extant elephants^25^. African elephants tend to have four toes on their forefeet while Asian elephants have five toes on the forefoot and four on the hindfoot (Panagiotopoulou et al.^26^, and references therein).

## Results and discussion

### The MTS proboscidean tracks and trackmakers

**Figure 2:**
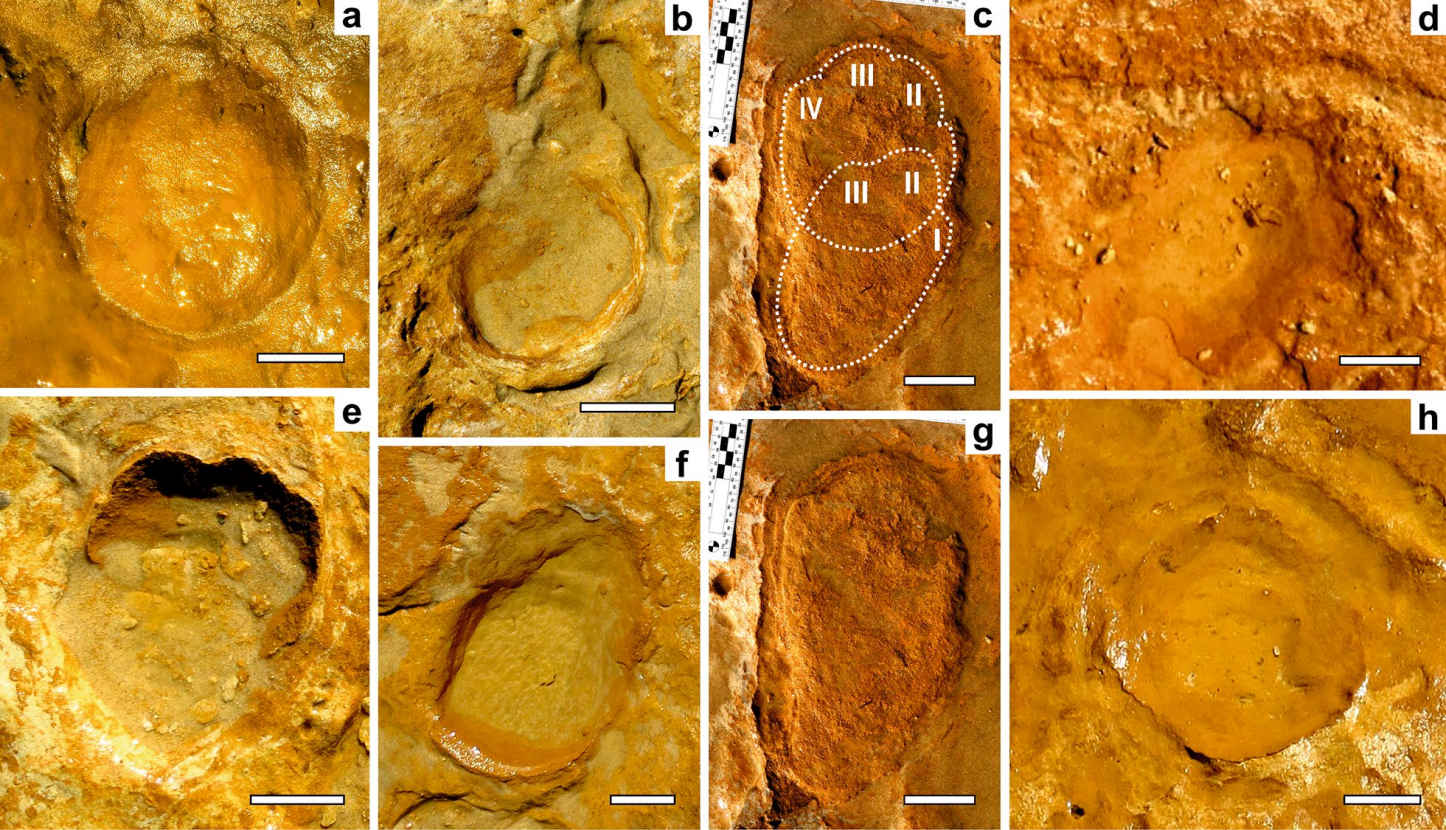
Proboscidean tracks (*Proboscipeda panfamilia*) attributed in the MTS to straight-tusked elephants. (**a**–**h**) Morphological features of small-sized tracks produced by calves and juveniles. Examples of *manus* impressions in (**a**) PAT/MTS/011a, (**b**) PAT/MTS/016 and (**f**) PAT/MTS/015x, and for further interpretation of (**a**) see Fig. 3; the latter two with drag marks made during the foot-off event. (**c**) and (**g**) PAT/MTS/002a,b: *Manus-pes* couple found isolated showing heteropody and different number of toe impressions (interpretation as left-side tracks by peak pressure deformation in the left side of the track according to^27^); interpretation in (**c**). (**d**) PAT/MTS/014 and (**e**) PAT/MTS/007a: Calf-sized *pes* with three toe impressions. (**h**) PAT/MTS/011 h: Badly preserved *manus* of a calf. Scale bar = 5 cm.

**Figure 3:**
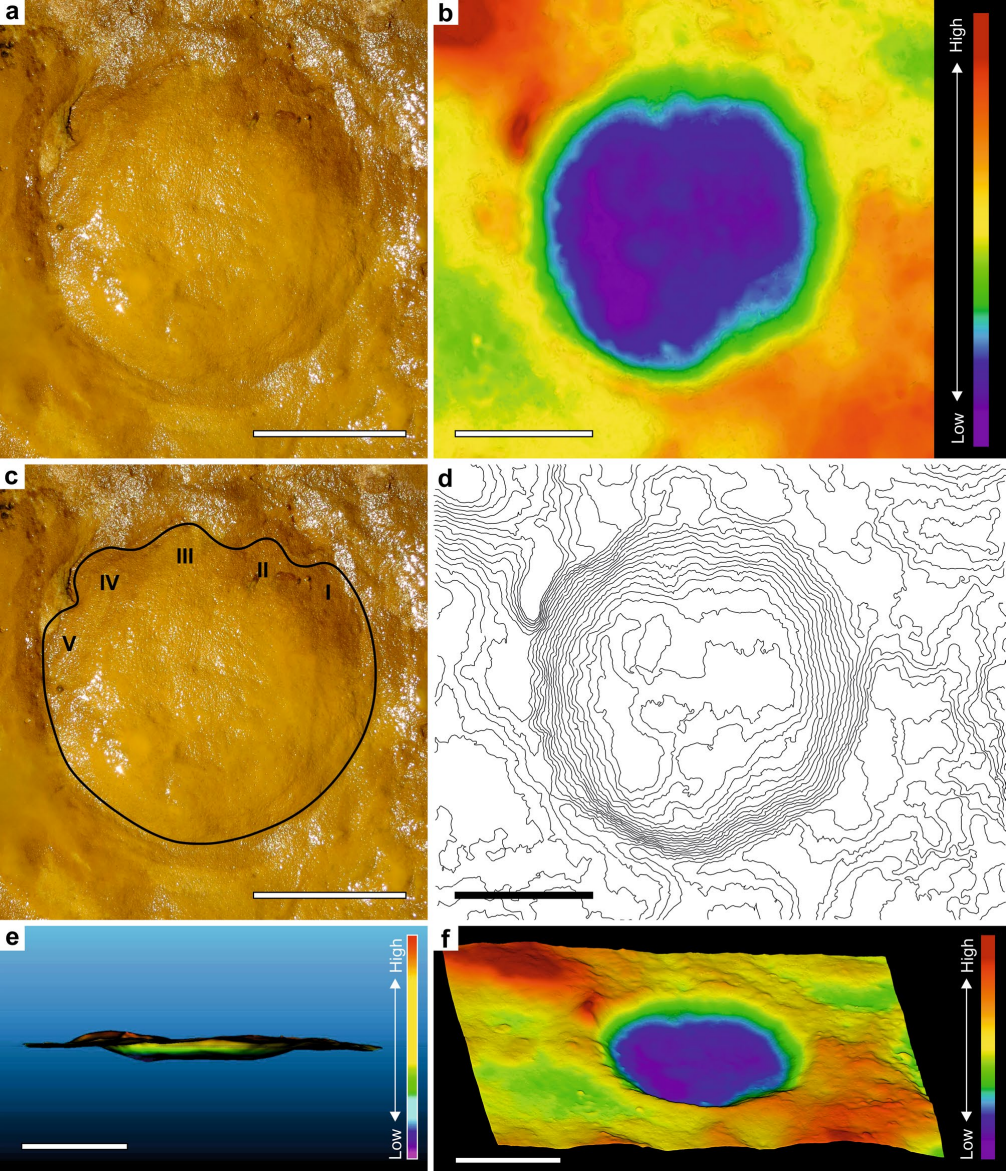
Photograph, outline, high-resolution 3D and false-coloured 3D images of the PAT/MTS/0011a track representing the best preserved *manus* of a juvenile-sized *Proboscipeda* track. (**a**) and (**c**) From the photograph and high-resolution images, five toe impressions in the anterior part of the rounded track are clear (especially toes I–IV). (**b**) and (**f**) The false coloured images in orthogonal (**b**) and oblique angle views (**f**) highlight the deepening of the track fore- and outwards, thus revealing a peak pressure pattern typical of left forefoot (toes III–IV), as well as a possible ejecta mound in front of the track. The poorly evident and narrow expulsion rim developed around the track is the result of the high cohesiveness and plasticity of the clayey fine-sand substrate. (**d**) Contour map supporting previous interpretation. (**e**) The cross-section of the track details the anterior migration of the foot pressure during its rotation, creating a peak pressure in the foot-off event that is represented in the deepest part of the track. Scale bars are 10 cm.

**Figure 4:**
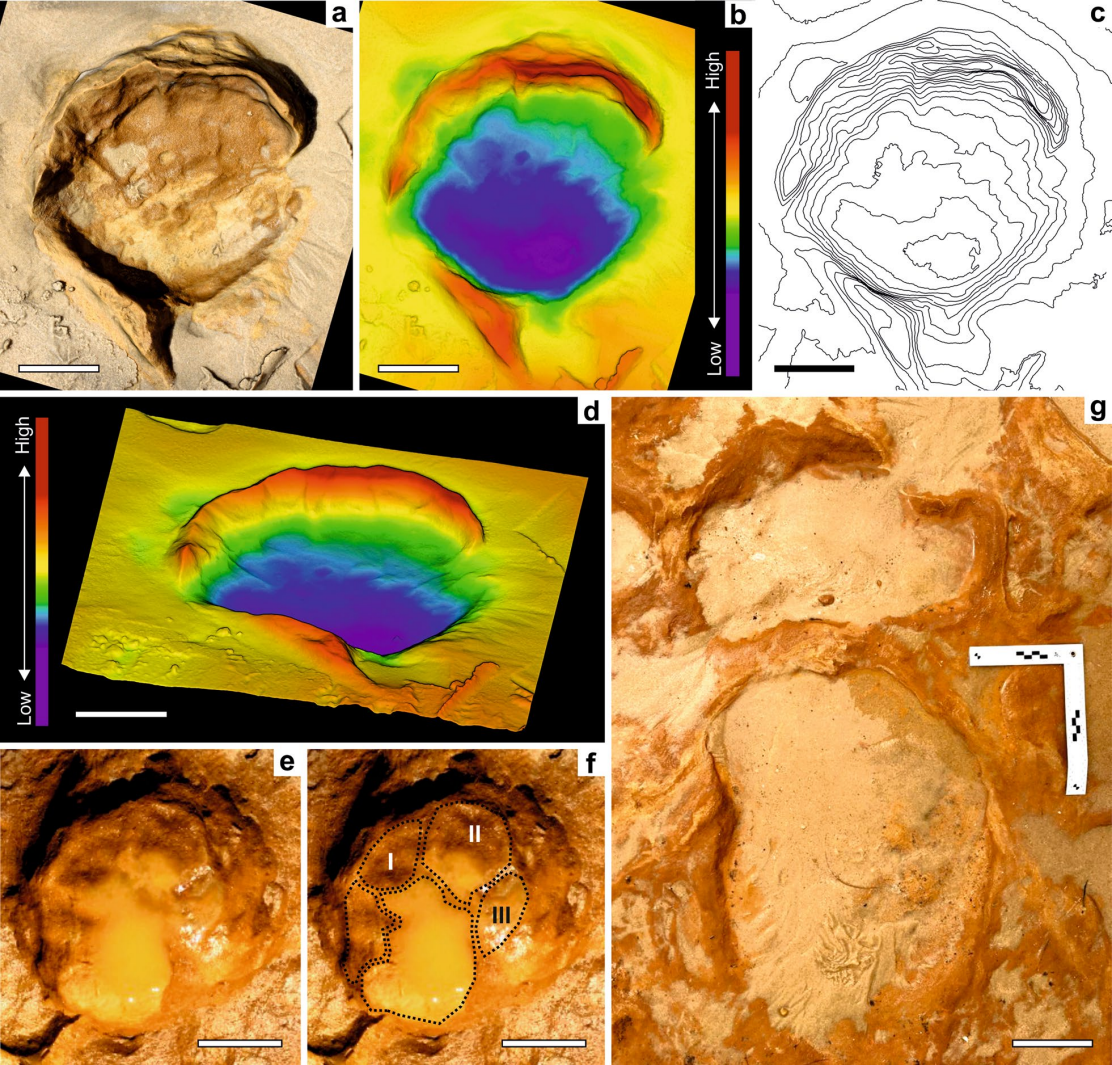
Large-sized *Proboscipeda* tracks attributed to *P. antiquus* adults. (**a**) to (**d**) PAT/MTS/001: Right *manus* showing clearly 5 toe impressions and the frontal and lateral displacement rims (morphological interpretation based on the orthogonal (**b**) and oblique (**d**) depth and contour (**c**) maps). (**e**) and (**f**) PAT/MTS/010e: Deeper manus with pockmarks; toe pad impressions indicated (I–III). (**g**) PAT/MTS/004a,b: large manus-pes couple where the hind foot deformed the fore foot during overstepping, and revealing a typical elephantine gait; the toe impressions in both tracks indicate the direction of movement. Scale bar = 10 cm.

Rounded-to-elliptical tracks, with an axial length range from 9.6 to 54.5 cm (*pes*), were found mostly isolated and as *manus-pes* couples, or associated forming at least eight short trackways (see Table 1). They reveal good preservation in one 6-footprint trackway (see below), two converging trackways and some couples, showing anteriorly directed, wide, short and blunt toe impressions (Figs. 2, 3 and 4). Toe impressions are not commonly visible in elephant footprints^9,13^, (but see^27^), which attests to cases of exceptional preservation in Matalascañas tracks. Preservation as true tracks is identified through expulsion marginal rims (e.g., Fig. 4a, g) and possible ejecta (Fig. 3b,e). Large and flat sole surfaces sometimes show evidence of pockmarks^23^ (Fig. 4f).

**Table 1:** Measurements of *Proboscipeda* tracks, ordered from smallest to largest in length.

Track no	*Manus Pes*	Length (cm)	Width (cm)	Toe impressions	E.S.H. (cm)	E.B.M. (kg)	E.A. (years)
**CALVES (< 2 years)**							
PAT/MTS/005	P	9.6	7.3		66 (m)	70 (m)	< 1 (m)
					56 (f)	43 (f)	< 1 (f)
PAT/MTS/012x	M	9.6	8.1		66 (m)	70 (m)	< 1 (m)
					56 (f)	43 (f)	< 1 (f)
PAT/MTS/016	M	10	8.2		69 (m)	79 (m)	< 1 (m)
					58 (f)	48 (f)	< 1 (f)
PAT/MTS/011a	M	10.8	11.7	5	73 (m)	93 (m)	< 1 (m)
					62 (f)	58 (f)	< 1 (f)
PAT/MTS/012j	P	11.3	9.4		76 (m)	105 (m)	< 1 (m)
					65 (f)	67 (f)	< 1 (f)
PAT/MTS/008d	M	13.8	13.7	4	91 (m)	177 (m)	< 1 (m)
					78 (f)	113 (f)	< 1 (f)
PAT/MTS/012a	P	14.1	10.8	2–3	92 (m)	182 (m)	< 1 (m)
					80 (f)	122 (f)	< 1 (f)
PAT/MTS/011f	M	14.2	11.5		92 (m)	182 (m)	< 1 (m)
					80 (f)	122 (f)	< 1 (f)
PAT/MTS/007a	P	14.6	10.5	3	95 (m)	200 (m)	< 1 (m)
					83 (f)	135 (f)	< 1 (f)
PAT/MTS/010f	M	15	17		98 (m)	219 (m)	< 1 (m)
					85 (f)	145 (f)	< 1 (f)
PAT/MTS/014	P	15.5	12	3	100 (m)	232 (m)	< 1 (m)
					88 (f)	160 (f)	< 1 (f)
PAT/MTS/003k	M	15.7	22.7		102 (m)	246 (m)	< 1 (m)
					89 (f)	166 (f)	< 1 (f)
PAT/MTS/015x	M	16	17		104 (m)	260 (m)	< 1 (m)
					92 (f)	182 (f)	< 1 (f)
PAT/MTS/012c	P	16.9	11.4		109 (m)	298 (m)	< 1 (m)
					95 (f)	200 (f)	< 1 (f)
PAT/MTS/012b	P	17.7	10.5	1	113 (m)	331 (m)	1 (m)
					100 (f)	232 (f)	1 (f)
**JUVENILES (2–7 years)**							
PAT/MTS/009	M	19.4	17		123 (m)	424 (m)	2 (m)
					109 (f)	298 (f)	1 (f)
3PAT/MTS/011e	P	20.6	15.3	3	130 (m)	497 (m)	3 (m)
					116 (f)	357 (f)	2 (f)
PAT/MTS/003g	M	21.8	23.8		137 (m)	579 (m)	2 (m)
					122 (f)	414 (f)	2 (f)
PAT/MTS/010b	P	22	17		138 (m)	592 (m)	3 (m)
					123 (f)	424 (f)	3 (f)
PAT/MTS/008e	P	22.6	15.1	3–4	142 (m)	643 (m)	3 (m)
					127 (f)	465 (f)	2 (f)
PAT/MTS/003i	M	23.4	23.1		146 (m)	697 (m)	3 (m)
					131 (f)	509 (f)	3 (f)
PAT/MTS/003a	M	26.5	31		164 (m)	976 (m)	6 (m)
					148 (f)	725 (f)	4 (f)
PAT/MTS/013	P	29.5	24		182 (m)	1321 (m)	7 (m)
					164 (f)	976 (f)	7 (f)
**ADOLESCENT (8–15 years)**							
PAT/MTS/003p	M	28.9	26.6		178 (m)	1238 (m)	8 (m)
					161 (f)	925 (f)	6 (f)
PAT/MTS/005	M	30	31		185 (m)	1385 (m)	8 (m)
					167 (f)	1029 (f)	7 (f)
PAT/MTS/001	M	30.9	33	5	190 (m)	1497 (m)	11 (m)
					172 (f)	1121 (f)	8 (f)
PAT/MTS/015a	M	31.1	32		173 (f)	1140 (f)	8 (f)
PAT/MTS/003d	P	33.3	24.5		204 (m)	1840 (m)	11 (m)
					185 (f)	1385 (f)	9 (f)
PAT/MTS/003b	P	37.2	27.8		227 (m)	2509 (m)	14 (m)
					206 (f)	1893 (f)	13 (f)
**ADULTS (> 15 years)**							
PAT/MTS/003j	P	40.1	21.8		243 (m)	3057 (m)	17 (m)
					222 (f)	1121 (f)	21 (f)
PAT/MTS/003c	P	40.7	29		247 (m)	3026 (m)	17 (m)
					226 (f)	2477 (f)	26 (f)
PAT/MTS/003f	P	45.8	30.7		277 (m)	4471 (m)	33 (m)
							> 40 (f)
PAT/MTS/004b	P	51	36.5	3	307 (m)	6027 (m)	< 60 (m)
PAT/MTS/003q	P	54.5	31.6		325 (m)	7111 (m)	< 70 (m)

*Pes* measurements were preferred in trackways but due to the relatively small sample of different sized-producers, *manus* dimensions were also included, especially in isolated tracks.

PAT/MTS—*Palaeoloxodon antiquus* tracks/Matalascañas Trampled Surface, a, b, c, …—representing tracks with different sizes/individuals in the same exposed area, M—*manus*, P—*pes*, E.S.H.—estimated shoulder height, E.B.M.—estimated body mass, E.A.—estimated age, m—male, f—female.

Irrespective of the track size, *pes* are elliptical to sub-rounded, with the length axis larger than the width and *manus* are circular or elliptical, with the width axis larger than the length (Figs. 2c and 4d, g for small and large size tracks, respectively). The safest way to differentiate between *pes* and *manus* is through the orientation of the track provided by the toe impressions, or by the orientation of the longer axis in trackways. When arranged in trackways, *manus-pes* couples show the typical elephantine gait, showing a short pace resulting from the fore- and hind feet on the same side swinging forward simultaneously below the body, as it is known from modern elephant gait^28^. In some cases, the partial impression of a *pes* overstepping the proximal part of a *manus* can be seen (Fig. 2c, g). Based on similar preservational style and opposing directions of movement without overlapping at the meeting point, a converging pair of trackways was apparently produced contemporaneously by an adult and a rather small juvenile. Sharp edges of the toe impressions indicate the presence of nails. These are found mostly in well preserved, smaller-sized tracks (Fig. 2a, d, e) because nails are commonly worn down in adult elephants and not always shown in their tracks^13^. These morphological features allow us to attribute the MTS trackways to the ichnospecies *Proboscipeda panfamilia* used previously for describing, among other tracksites, those tracks attributed confidently to the straight-tusked elephant *Palaeoloxodon antiquus* in the paleogeographical context of southern Europe^11,14^ (see supplementary Table S1).

*Manus-pes* couples, when showing overstepping, were not considered in Table 1 (Fig. 2c, g). Overstepping depends on the speed of walking; at faster speeds the overstepping is only partial or there is no overstepping; elephants maintain the footfall pattern at all speeds, shifting toward a calculated 25% phase offset between limbs as they increase speed^28^ (Fig. 2g). The smallest tracks usually do not show overstepping possibly because of the greater activity, with longer pace and stride lengths, demonstrated by calves and juveniles when compared to adults. *Manus* or *pes* showing a large width-length ratio (below 0.80–0.96 sensu^25^) were not considered for the estimates since they represent slippage.

Younger elephants have more pliable skin and musculature than adults. Also, the greater expansion and distribution of the weight in heavier adult animals is enough to reduce or negate toe impressions in some types of sediments, such as compacted substrates^24,29^. Interpreting the sedimentological data for the paleosol where MTS was developed^15,17,30^, suggests a drying clayey-sandy substrate^14^ that was still plastic enough to absorb the impact of the limbs during the locomotion of the elephants (presence of expulsion rims and absence of radial pressure cracks), and preserving, in many cases, the morphological details of the feet in good condition (Figs. 2a, 3, 4a; see Fig. 2h for a badly preserved example).

### Ichnological inference about the height, body mass and age of *Palaeoloxodon antiquus* in the MTS

Several methods have been proposed for estimating the height at the shoulders for proboscideans, and the relationship between body mass and age with shoulder height ^1,31,32^. A linear relationship between foot length and shoulder height was confirmed by Lee and Moss^33^ from extant elephants and compared with fossil examples by Pasenko^24^. *Pes* length has been especially used in studies as an indicator of shoulder height^21,34–36^. Among Asian elephants, *manus* circumference has been shown to have a similar predictive relationship with shoulder height^33^. These parameters were determined for each isolated track (or representative track in a trackway), including manus and pes (Table 1), using equations previously proposed^31,33^ (see Methods). A similar approach has been applied to mammoth track studies in North America^21,27^, where modern ontogenetic and body-mass data has been used to provide age and size estimates from fossil tracks.

From the skeletal record, sexual dimorphism of *P. antiquus* was observed to be more accentuated than in extant elephants, especially in terms of size differences^1^. During the first 10 years of life, both male and female African bush elephant foot lengths increase rapidly, with the fastest growth shown in the first two years for calves^33,37^. In *P. antiquus*, males would have continued to grow until their fifties according to bone data^1^, while females would have been much smaller as result of energy expenditure with reproduction, flattening the growth curve just after puberty. That is why the equations of Lee and Moss^33^ that discriminates the shoulder height from tracks for males and females have been applied. However, by comparison with the study of Marano and Palombo^32^ (based on the progress of eruption and degree of wear of teeth compared to extant elephants), and the body mass correlation of Larramendi et al.^1^ for calculating the age of *P. antiquus*, our MTS ages obtained from the application of the regression curve of Lee and Moss^33^ are underestimated and must be analysed as minimum age approximations for track lengths corresponding to adolescent and adult animals, especially for males. The obtained estimations from tracks are subject to a level of uncertainty related to biotic and abiotic factors that can distort the data (i.e., taphonomy) as it happens also with the calculations taken from skeletal proportions. Therefore, McNeil et al.^21^ even included data from frozen mammoth carcasses on the growth curve of Lee and Moss^33^ for correcting size discrepancies along ontogeny. For *P. antiquus*, our best data for comparison comes, however, from the flesh reconstructions^1^.

### Ontogenetic implications

Based on the best fossil site found for this species in Europe, corresponding to 70 individual *Palaeoloxodon antiquus* specimens recovered in Geiseltal, Germany, Larramendi et al.^1^ developed the best reconstruction, so far, of the life appearance of this species and discussed size, body mass, ontogeny and sexual dimorphism. The Neumark-Nord bone site may be contemporary or slightly older than MTS, corresponding to late Middle Pleistocene-to-Eemian interglacial period^1^. The authors found that the body mass of *P. antiquus* males was up to three times more that of male Asian elephants and twice that of extant male African bush elephants. The large size determined for straight-tusked elephants (with an estimated > 400 cm shoulder height in the flesh and body mass of 13 tonnes) and a later complete epiphyseal-diaphyseal fusion of limb bones (not yet totally fused at an estimated age of 47 years), in comparison with extant elephants, suggests that this species had a longer lifespan of 80 years or more^1^. Sexual dimorphism of *P. antiquus* was observed to be more accentuated than in extant elephants, with females generally not exceeding 300 cm at the shoulders with an estimated weight of not more than 5.5 tonnes, while males continued to grow until their fifties^1^. Males in extant elephant species grow more rapidly than females after puberty (i.e., around 7 years in age), which are affected by a trade-off between growth and reproduction. Under normal nutritional conditions, the growth rate is generally higher in males than females leading to a marked difference in size between sexes at already around 10 years in age^33,37–39^.

**Figure 5:**
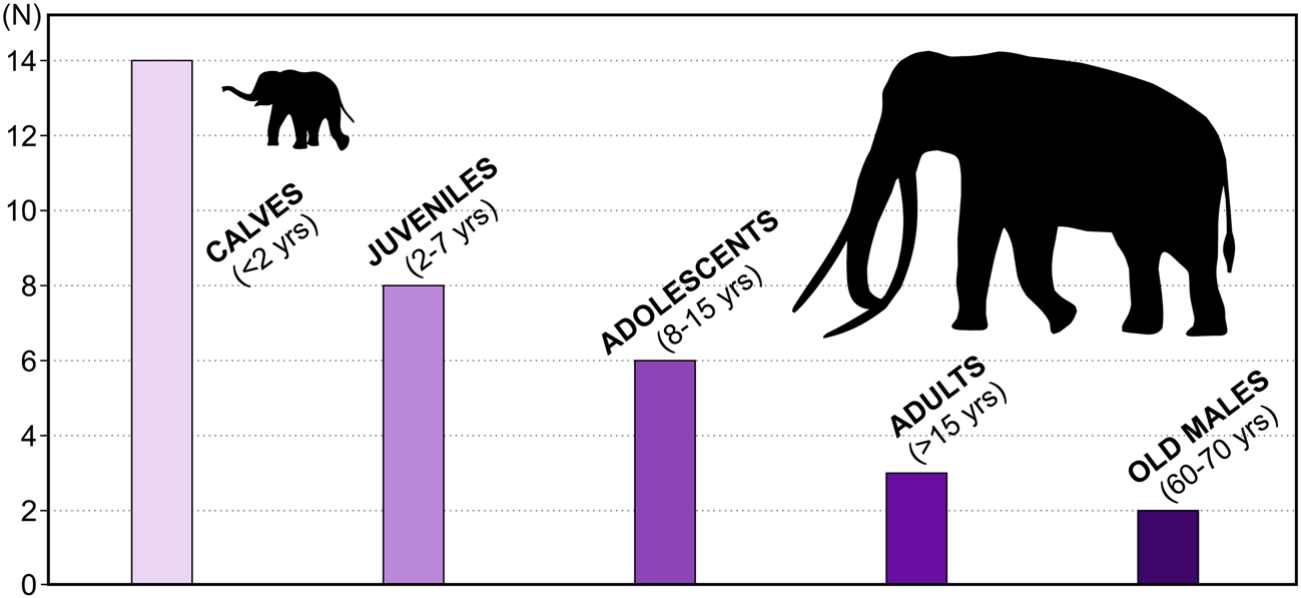
Demographic frequency plot determined from *P. panfamilia* track measurements in MTS. (N) represents the projected number of *P. antiquus* individuals measured from track (mostly *pes*) size (see Table 1).

**Figure 6:**
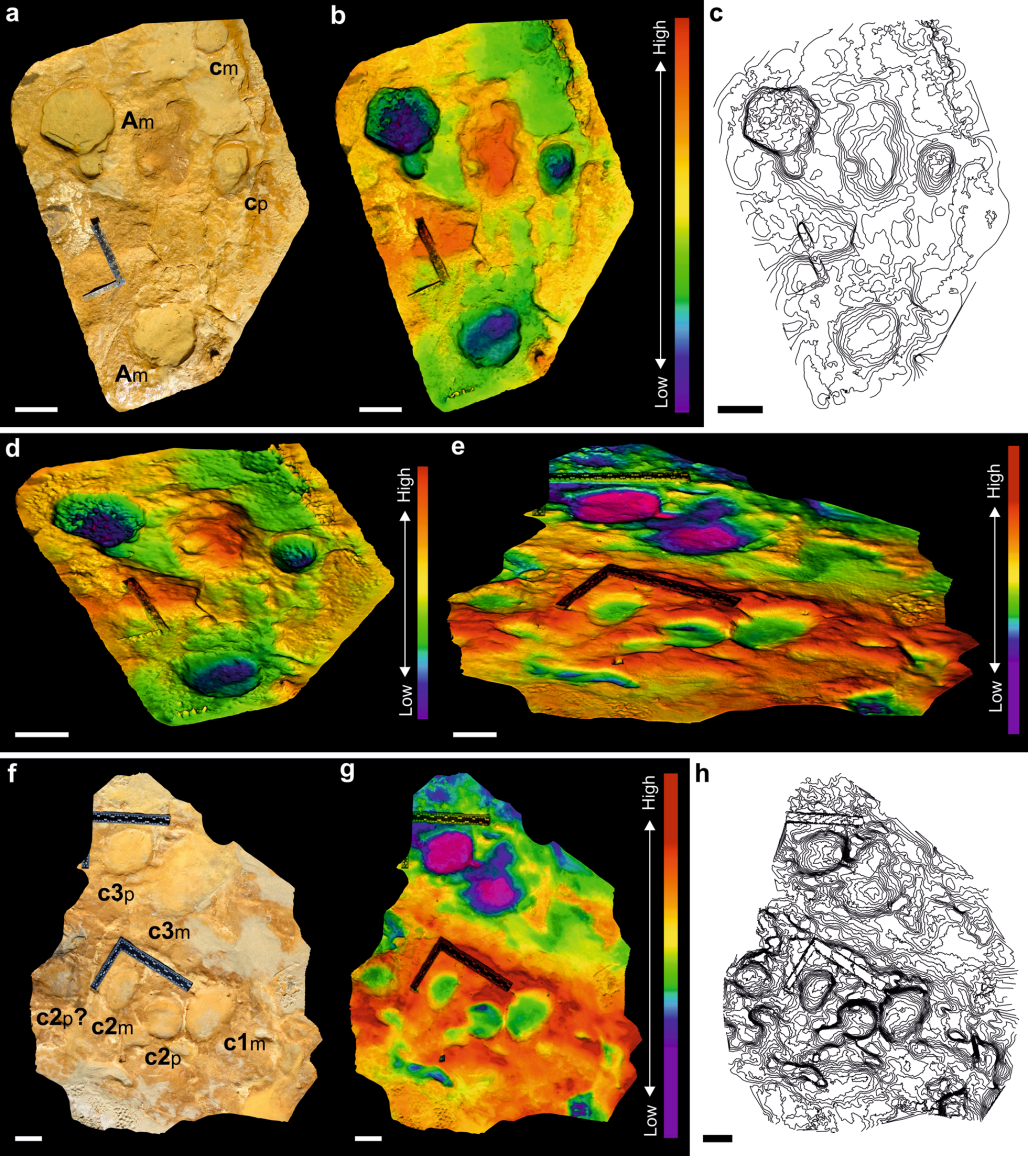
Examples of adult (presumably female)-calf trackways (PAT/MTS/015: for measurements of tracks and estimations of body mass and age see Table 1). (**a**) Real-image of two parallel trackways showing the same direction of movement indicated by the presence of drag marks in the calf trackway and the deepest part of the track always located in the same size of both trackways (interpreted as the anterior one) as shown in the vertical (**b**) and oblique (**d**) depth maps and the contour maps (**c**). Scale bar = 20 cm. (**e**–**h**) PAT/MTS/008: Tracks of young elephants produced contemporarily except the two larger ones which are deeper imprinted (scale bar = 10 cm).

**Figure 7:**
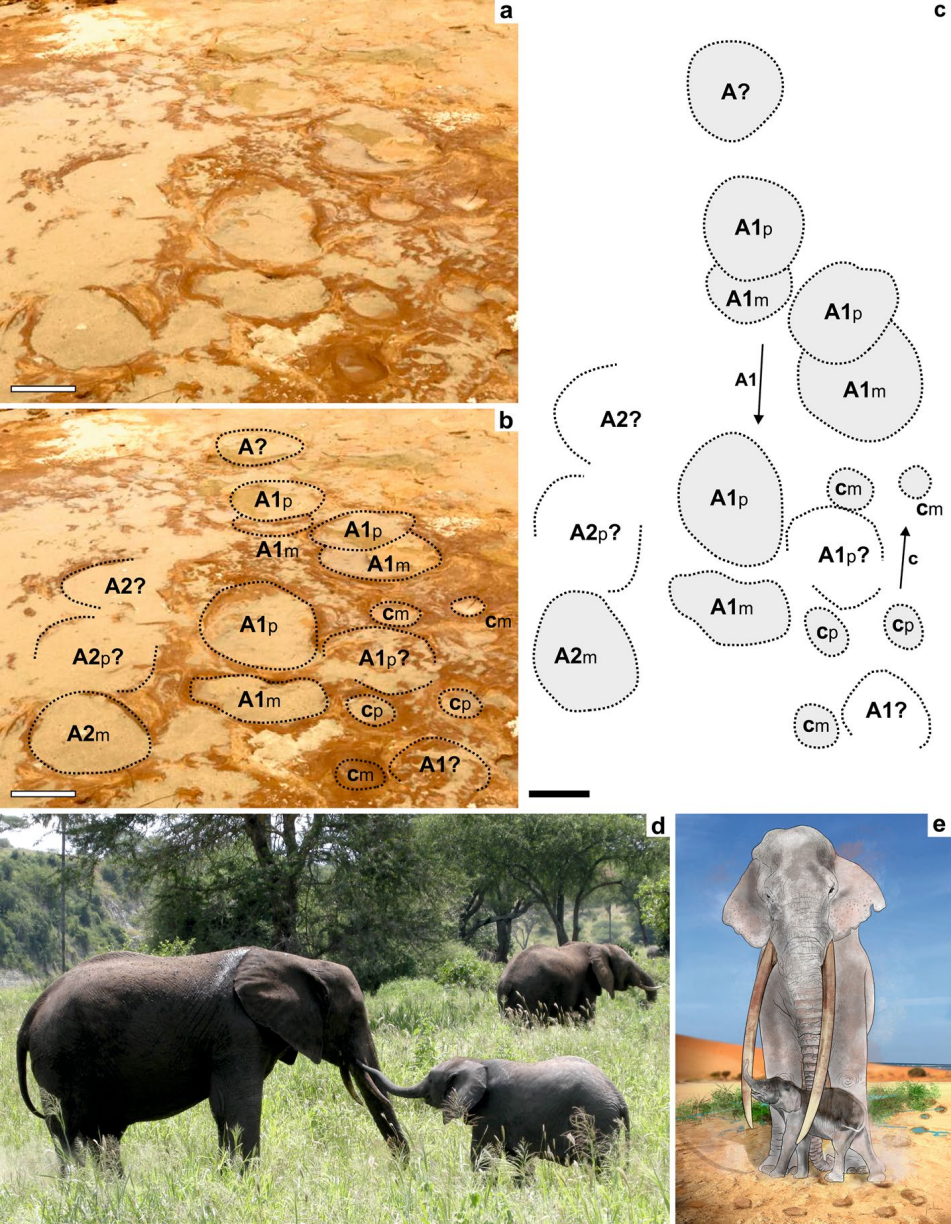
Ichnological evidence and reconstruction of *Palaeoloxodon antiquus* social interactions deduced from the MTS. (**a**–**c**) Two adult (presumably female) ‘A1 & A2’ and one juvenile trackway ‘c’ showing convergence (the toe impressions indicate opposite orientation of movement); note overstepping of *pes* over *manus* in the main adult trackways that is not seen in the smaller tracks, in this case because the small juvenile may have stopped just after the larger animal slowly passed by (interpretation in **c**). (**d**) Example of a young mother-newborn *L*. *africana* interaction. (**e**) Reconstitution of mother-newborn interaction in the MTS (artwork of J. Galán).

The ontogenetic variation in growth projected for the MTS, when compared to what we known from extant proboscideans, is expressed in the track size distribution plot, with the definition of five age classes (Fig. 5; see also Table 1): calves under 2 years in age (when extant elephants experience fastest growth rates in both sexes), juveniles between 2 and 7 years in age (up to when elephant females reach their sexual maturity and therefore experience a strong reduction of growth rate in comparison to males), 7–15 years in age which include pre-puberty males and young female adults, over 15 years in age and < 70 old bulls (with almost stagnation of female growth and males reaching much larger sizes). Trample grounds are important for identifying the social structure and interaction of groups of animals as they represent an extremely short period of time, and thus provide a snapshot of group behavior^21^. Thus, the MTS reflects the demographic structure of the *P. antiquus* present in this this habitat at the time it was formed. The age classes-frequency plot shows that a large majority of small tracks found belong to calves within one year in age, with estimated shoulder heights less than 95 cm (Figs.4, 6), and estimated body masses between 70 and 200 kg (Table 1). The post-puberty gender distribution cannot be reflected by the tracks both males and females could produce, due to the fast growth of bulls in relation to females of reproductive age except, and with a certain confidence, when trackways of large and very small animals are found interacting or show parallel direction of movement (Figs. 5, 6 and 7). Only the largest, over 50 cm long tracks already identified in the MTS can confidently be attributed to males (old bulls), but the tracks around 30 cm or less may have been produced by adolescent males or young adult females. The evidence for large bulls, up to 325 cm of estimated shoulder height and over 7 tonnes of estimated body mass, are surprisingly very rare in MTS (represented by only two tracks) and may represent solitary incursions to this habitat (see below).

In the MTS, true tracks reveal the plantar-contact surface. Depth distribution within the track reflects the applied plantar pressure on the thick pad, in all age groups. Living elephants usually touch down phase with their heels and step off of their toes^29^. Patterns of depth-deformation for *P. antiquus* from MTS are consistent with the plantar pressure data for modern elephants where, for similar substrate conditions, there are no significant depth differences between animals with estimated body masses of 260 kg and over 1 ton (Figs. 6a–d, 7a). The deepest zone of the track corresponds to its anterior part following the typical rotation during the event foot on-toe off observed in extant elephants (see Fig. 3b, e for a MTS example). The pressure on the feet of elephants is greater on the distal ends of the lateral toes and increases outward from the centre of the foot^24,26,40,41^. This may leave prominent anterior nail impressions in the plastic substrate of Matalascañas for contemporary small and large tracks.

Several of the small- and large-sized proboscidean tracks have been produced contemporaneously in the MTS (Fig. 6a–d). However, foot length for age in Fig. 5 reveals the preferential presence of young *P. antiquus* in the MTS, especially calves and juveniles. Smaller elephants are relatively more agile than larger ones, but still move with the same gait as adults^28,36^. For that, they just need to adopt greater relative stride frequencies and relative stride lengths compared to larger elephants^21^. That is the reason why overstepping is not evident (but see Fig. 2c for an exception found) in the juvenile-sized trackway but is remarkably evident for the adult-size trackway which intersects it (Fig. 7a–c). Smaller animals took quicker steps with shorter contact durations with relatively longer pace lengths. For this reason, the good preservation of morphological features, without being blurred by overstepping or confounded with similar-sized tracks of other mammals especially in less preserved examples, enabled to discriminate many small proboscidean-type tracks. Their attribution to several individuals based on track size comparisons reflect the prevailing and contemporary presence of youngsters during the short-term environmental conditions that allowed the development and preservation of the MTS.

### Matalascañas as a possible straight-tusked elephant reproductive habitat

Extant elephants possess a sex-segregated social structure, centred around a matriarchal family, consisting of one or more related females and their offspring. Males are raised until sexual maturity, around 14–15 years in age, when they approach the shoulder height of adult females^42^, then becoming loners and only reuniting with the female-led groups for mating^43^. Nuclear family structure in *Loxodonta cyclotis* corresponds to a mother-calf pair, or 2–4 animals, and herds in *Loxodonta africana* are usually extended families between 4 and 14 animals^42,44^. African forest elephants’ males are solitary, while making transient associations in *Loxodonta africana*. The remarkable rarity of very large proboscidean tracks in the MTS, with only two tracks over 50 cm in length and clearly made by old bulls, suggests the possibility that the area was visited mainly by matriarchal groups. The characteristics of the MTS as event surface and the comparison of track preservation conditions, such as depth and deformation structures in a relatively firm substrate^45^ (Fig. 6) provides data to define the impression window for different trackways and tracks which support herding behavior for *P. antiquus*.

In the original diagnosis of *Proboscipeda*, Panin and Avram^46^ suggest likely herding behavior for a large number of deinothere tracks preserved on a small surface, from the upper Miocene of Romania. As in MTS, Mleisa 1 in Abu Dhabi preserves evidence for a herd of *Stegotetrabelodon* Petrocchi of varying size crossed by single large animals indicating the presence of both herding and solitary social males^43^. The presence of a small individual around 1 ton in estimated body mass was inferred from these tracks. A herd of probable *Palaeoloxodon recki,* consisting of both adults and juveniles trackways measuring 19-to-63 cm was described in MIS 5 paleolake deposits from the Arabian interior^47^. Gregarious behavior and matriarchal herding were previously known for straight-tusked elephants from their track record in similar-aged, MIS 5 eolianites from SW Portugal^11,48^. There, a trackway attributed to a juvenile was found parallel to other same-directed trackways considered to be two adult females^48^. A trample ground in the same coastal eolianite reveals a concentration of different sized tracks from where very large asymmetric undertracks organized in parallel trackways, and interpreted as produced by large males, seem to diverge^11^. This interpretation of social herding structure for *P. antiquus* seems to be different than the one in MTS, most likely because of a distinctive habitat use.

Matriarchal herd behavior among proboscideans is not surprising and likely evolved early, with its earlier records dated from Late Miocene^43,46,49^, being also deduced from tracksites in the latest Late Pleistocene of both *Mammuthus primigenius*^20,21^ and *Mammuthus columbi*^12,22,27^. The presence of calves in these herds was demonstrated only in very rare and remarkable cases. Retallack et al.^12^ described a trample ground of *Mammuthus columbi*, from the shores of Fossil Lake in Oregon, and dated from 43.26 ± 0.33 cal ka, showing a herd of 4 adults, a baby, and at least one subadult. The smallest trackway estimates the presence of a calf less than a year old, and intermediate sizes between 1 and 3 years old. In New Mexico, Lucas et al.^22^ described a likely (Columbian) mammoth tracksite of Late Pleistocene age (22.8–19.43 ka) with hundreds of proboscidean tracks from 15 to 62 cm in length, estimate shoulder heights ranged between 180 cm (juveniles) to 300 cm. Matsukawa and Shibata^49^ described a tracksite produced by a herd of *Stegodon aurorae* in the Late Pliocene of Tamagawa. Two parallel trackways of large and small proboscidean tracks suggest a female adult and juvenile. In the MIS 5e-to-5b eolianites from Still Bay, South Africa, coeval with the MTS, *Loxodonta africana* tracks were described in heavily trampled surfaces, suggesting the passage of a sizeable herd of elephants^50^. Tracks with the smallest length of 17–19 cm were made by calves about 1 year old, found in two parallel trackways made by two very young animals, walking side-by-side, and influenced by mutual behaviour.

In conclusion, herd structure and social behaviour have been previously inferred for the extinct gomphothere *Stegotetrabelodon,* deinotheres, mastodon^51,52^, *Stegomastodon* and *Stegodon aurorae* from Japan^49^, Columbian and woolly mammoths, *Loxodonta africana, P. recki* and *P. antiquus*, from upper Miocene and Pleistocene trackways and also from mass-death assemblages^53^. The MTS provides detail to the existing knowledge on the social structure of *P. antiquus*, with the evidence of matriarchal groups with newborns, perhaps with just few days to months of existence (Fig. 6), and the inferred rare presence of adult males.

African bush elephants have a hind footprint length at birth of 12.5 cm and a weight of 90 kg, with a minimum estimated shoulder height of 69 cm from a young calf^42^. For the Amboseli *Loxodonta africana*, the minimum shoulder height measured was 79 cm for a newborn female^33^. A comparison of the size categories of *Loxodonta cyclotis* with those of *Loxodonta africana* suggested a similar distribution of size, despite a marked difference in stature^42^. However, growth may have occurred more quickly in *P. antiquus*, as recorded in the MTS, than for *Loxodonta africana*, similar to what is known for mammoths^20^. The Yamal baby mammoth male had a hind foot of 10 cm in length and 8 cm in width, with a measured shoulder height of 67 cm and an estimated age of 3–4 months^54^ and therefore newborn *P. antiquus* tracks of the same size, or slightly shorter, from the very plastic sediments of the MTS may have been made by animals with equivalent shoulder height and body mass, i.e., up to 66 cm and around 70 kg.

Female-led family groups are required to remain closer to water as the young need to drink more frequently and cannot cover large distances as quickly as the adults. This tends to restrict the nurseries to areas close to available water^20^. Therefore, the MTS seems to have been a prime area for matriarchal herds as the bias towards the inferred presence of newborn and juveniles is noteworthy. We interpret the trackways of Figs. 5 and 7a as two examples of straight-tusked elephants walking together, most likely a female moving slowly in response to a close juvenile (reconstitution in Fig. 7e).

The dietary behaviour of *P. antiquus* populations in different climatic conditions, resulting from microwear and isotopic analysis^55^, has documented different feeding behaviours, particularly browser versus grazer-prevalent diets. African forest elephants living in moist semi-deciduous forests and rainforests are browser-frugivorous^44^. However, high numbers of elephants use the coastal area of Gabon, with strongly seasonal rainfall, throughout the year^56^. Elephants were observed foraging along the coastal shrub areas adjacent to the beach, particularly during the rainy season. Seasonally they spend more time in grassland than in forest during the short-wet season, when grasses grow faster^57^. Reolid et al.^6^ hypothesized that in southern Spain, the *P. antiquus* would have seasonally migrated beween the highlands of the Guadix-Basa Basin and the lowlands of the western Guadalquivir Basin where the MTS is located. As in the coastal area of Gabon, the MTS coastal area may have provided a mosaic of open grasslands in the dune complex and evergreen forest with high tree and shrub density near the banks of the Guadalquivir river, that afforded cover and browsing to the matriarchal groups during the Last Interglacial. Straight-tusked elephants may have included fresh grasses from the humid grassland dependent on the interdune ponds, in their diet. In the African elephants, mating usually occurs during the dry season^58^. If the gestation in *P. antiquus* was expectadly like for any modern elephant, i.e., around 20–22 months^59^, this would imply giving birth during the spring after the rainy season, when the freshwater ponds in Doñana would be in their full capacity, as in present days^60^. The retreating waters, starting in May, would have left behind important grasslands that may have provided available water and high-nutritious food resources for the new mothers and breast-feeding calves. The paleosol development with abundant rhizoliths clearly shows the growth of dense vegetation. The high-plasticity of the trampled clayey-sandy substrate and the presence of shrinkage cracks intersected by some tracks allow us to conclude that the water-level was retreating in the MTS interdune pond. The rare occurrence of large tracks shows that MTS was not a habitat for the male adults of *P. antiquus*. Nevertheless, the Doñana interdune freshwater pond system is an important source of available water in the coastal area and the pond-dependent seasonal grasslands would have been available, especially for old males requiring vegetation that was easier to chew.

### Neanderthals in the MTS: food procurement and elephants

**Figure 8:**
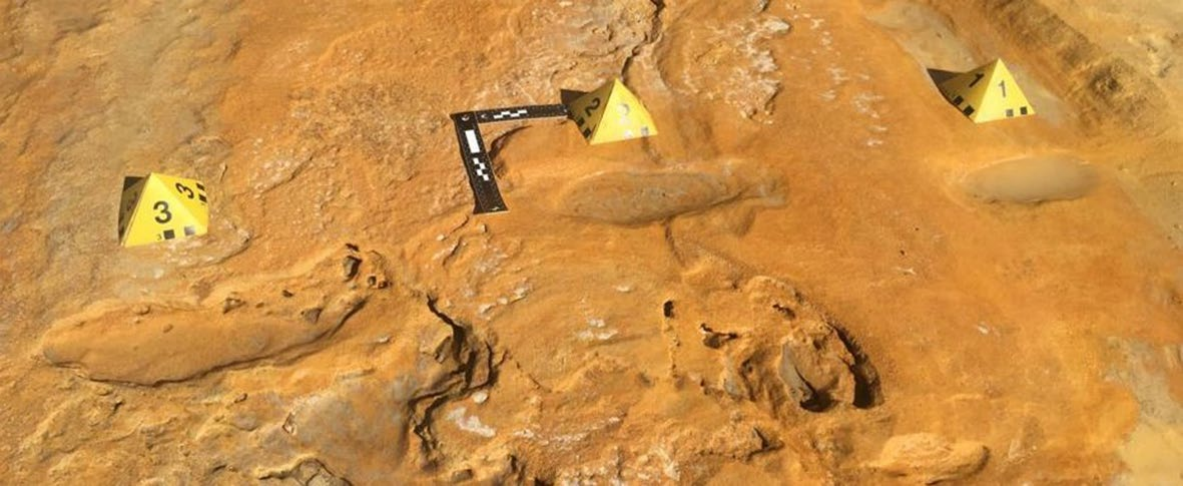
Hominin trackway attributed to a neanderthal adult found in an exposed area of the MTS (see the large well-preserved cervid track in the bottom of the photo). While track 3 still shows several details such as digit impressions and the expulsion rim in its outer part, tracks 2 and 1 were progressively eroded by the tides and lost their morphological features, including the shallower parts of the tracks, becoming unrecognizable.

During the study of the MTS, short trackways and isolated tracks of both adult and young humans were found together with large herbivore ones (Fig. 8). Meanwhile, Mayoral et al.^18^ claimed to have found 87 hominin tracks, most of them constituting what they called the *Hominin Trampled Surface* (HTS)*.* However, it is evident from their Fig. 3, that HTS is the continuation of the MTS in the most exposed area of the present shore to tidal and wave erosion. This important finding rises questions regarding the ecological reasons for the presence of Neanderthals in coastal environments and especially in the MTS. In fact, reconstructing hominin paleoecology is critical for understanding diets, social organizations and interactions with other animals^6^.

During MIS 5, Neanderthals would find in the coastal areas of SW Spain, visited by large herbivores according to both the track and body fossil (including *P. antiquus*^61^) record, important food resources for hunting and scavenging^62^, and the opportunity of including seafood in their diets^63^. In fact, besides Neanderthal tracks in the MTS we recorded the presence in concentrations of Mousterian-type lithic tools which are still under study. Neanderthals may have actively selected the best body parts for meat and fat from mega-herbivores, including proboscideans. Elephants are relatively easy to locate due to their dependency on water resources, reported use of familiar paths, and the clear tracks they leave behind^64^. There is an increasing amount of evidence that proboscideans, and especially young individuals, played a major role in Neanderthal’s diet and adaptation^64–67^. Anzidel et al.^67^ interpreted a late Middle Pleistocene occurrence in Italy of *P. antiquus* with Neanderthal lithic tools as resulting from scavenging activity. Contemporary from Matalascañas, in Manzanares valley (Madrid), Panera Gallego et al.^68^ have found typical Neanderthal lithic tools associated to skeletal remains of a possible young straight-tusked elephant, including also a couple of tracks 17–22 cm in length that could be related to the same animal. Considering the ichnological record of elephant-human interactions, McNeil et al.^20^ described a woolly mammoth tracksite in southwestern Canada dated between 11,3–11 ka with ichnological evidence of modern humans hunting the megafauna. Fladerer^69^ in skeletal record and Haynes^53^ in the track record found selective mortality of juvenile mammoths as result of modern humans selecting the smaller animals because they were easier to kill or to process. Mammoths-human interactions were also revealed through tracks in New Mexico^27^.

The repeated pattern of young proboscidean procurement suggests that age played a significant role in their selection, likely related to a better nutritional value and the relative ease to hunt and butchering, as well as transporting the best nutritious parts^64^. Therefore, and in parallel with the examples provided, the coetaneous presence of Neanderthal tracks and lithic tools together with megafauna trackways, including *P. antiquus*, rises the hypothesis that the MTS coastal pond may have been an elephant resource habitat for Neanderthals. They would be here seasonally intentionally hunting more accessible targets such as calves, juveniles or weakened females in delivery, or opportunistically scavenging stillbirths and females dead from birth.

## Methods

Since the MTS is located in the intertidal zone of the Matalascañas beach, an area affected by mesotidal gradients (mean tidal range above 2 m^30^), and usually covered by beach sand (Fig. S1), the time factor constrained data recovery. Field work consisted largely in a daily survey for MTS areas cleaned from sand by the tides, taking measurements from tracks and trackways using standard vertebrate ichnological procedures, including GPS location, and taking photographs with a Canon PowerShot SX50HD and Canon EOS 1200D. Most of the time the MTS is almost fully covered with over 1.5 m of sand beach that prevents the full perspective and exposure of the MTS. Only an exceptional situation of the spring storm surges in 2020 allowed to make detailed analyses of a wide area of the tracksite in the backshore not exposed to continuous tidal erosion, and where well-preserved tracks can be found in the MTS.

Sedimentology of the paleosol unit, in which the trampled surface with proboscidean and other mammal and bird tracks was developed, was detailed by Zazo et al.^15,30^ and Roquero et al.^17^. MTS occurs on the top of the paleosol PS1^30^ at the base of the succession, and at the toe of the El Asperillo littoral cliff, which represents the Quaternary infill of the Neogene basin of the lower Guadalquivir River. Tectonic uplift and sea-level changes in the coastal plain allowed the accumulation of a Pleistocene-Holocene succession of stacked, weakly-cemented to uncemented eolian sand dunes. Sediments in this succession consist mainly of very well sorted, medium to fine sand. Quartz grains average 70%, plagioclase and potassic feldspar less than 5%^17^. The PS1 at the eastern end of the El Asperillo cliff, developed in a sedimentary hiatus within the OSL-dated AU1 eolian unit, is described^30^ as a unit “intensely burrowed, clayey sand with vertically elongated, redoximorphic features formed during a temperate, moist climate with a dry season”. Vertical structures, forming mottles with a high kaolinite-content (19%), are related to rhizoliths, and ferriargillans reveal clay illuviation related to hydromorphic processes, mostly in reducing environments around roots^17,30^. The hiatus was long, resulting in the development of a mature paleosol 1 m thick, under the influence of vegetation and a shallow phreatic level linked to the uprising sea-level during the last interglacial period^30^. The shallow phreatic level would frequently surpass the topographic level in the interdune area during the wet season, creating or recharging interdune lakes or ponds^14,16^. The top of PS1 shows evidence of erosional truncation^17^ which means that the paleosol was subjected to erosional phases, possibly during the dry season, that might have prevented the preservation of tracks. Therefore, during the last drying phase of the interdune lake, the MTS may have been formed. Preserved exposures of the PS1 were quickly covered by either a ferriargillan coating and by eolian sediments from the AU1, which allowed the preservation of the MTS from weathering and erosion.

For numbering of the tracks, we established sectors in the MTS with areas exhibiting proboscidean tracks, where the different track lengths could clear indicate different individuals required for the calculation of the population structure. Track size subdivision was based *grosso modo* on the main age classes defined for extant elephants that show sexual dimorphism especially after the juvenile period: calves (fastest growth period within the first two years of life), juveniles (the end of this period defined by female puberty), adolescents (the end of this cycle marked by male puberty), adults (a period where it is not possible to differentiate males from females in the track record) and old bulls (whose track size is larger than any track produced by females). The current application of the shoulder height on foot length equations^33–36^ in studies of both extant and fossil proboscidean populations allowed us to estimate body mass and age for the track producers in the MTS. Estimations with both *manus* and *pes* lengths were made since we found no significant difference when comparing results from the same trackway. Usually, the criteria used was to measure *pes* preferently, or manus if *pes* were not available (for trackways). The estimation of shoulder height, body mass and age followed formulas currently in use for studies of extant proboscidean populations distribution, except for body mass, whose equation was recently developed specifically for *P. antiquus* by Larramendi et al.^1,36^.

Therefore, for shoulder height (E.S.H.) on foot length (FL) we used the regression of Lee and Moss^33^,

$$\begin{aligned} E.S.H.\left( {male} \right) & = - 10.22 + \left( {5.816 \times FL} \right) \\ E.S.H. \left( {female} \right) & = 3.044 + \left( {5.466 \times FL} \right). \\ \end{aligned}$$

Body Mass (E.B.M.) for *P. antiquus*^1^,

$$E.B.M. = 3.63 \times 10^{ - 4} \times E.S.H.^{2.903}$$

Finally, the estimated shoulder height for age was plotted in the Van Bertalanffy fitted curves for both male and female *Loxodonta africana* defined by Lee and Moss^33^ to obtain an approximate age for the trackmaker.

For the morphological study of the tracks, we applied current standards of data acquisition and 3D image analysis using digital photogrammetry^70^. Photos were taken in orthogonal and oblique views (resolution 4000 × 3000 and 5184 × 3456 pixels, in JPG format). Scales were placed on the surface to subsequently assign metric accuracy to the 3D models produced. The photogrammetric processing of the tracks was carried out with the free open-source software OpenDroneMap 2.1.0^71^ and Meshroom 2021.1.0 (© 2010 2019 Alice Vision^72^. The post-processing was carried out using free open-source software MeshLab v2020.12^73^ and CloudCompare v2.11.0.^74^. Post-processing phase involved the analysis, treatment, fit to the plane, orientation and scale assignment to the textured photogrammetric models obtained. The attribution of techniques that allow the microtopographic enhancement of the tracks under analysis, substantially facilitate their interpretation and, consequently, the achievement of more precise measurements and angles, often allowing observation of details that, in fact, would not be easy or possible to observe in the field. For the purpose of interpretation and visualization of the new tracks presented, we preferred the presentation, in different views, of 3D models in false colours highlighted with shadows, representing the altimetric variation (depth maps) of the modelled surfaces. The Fig. 3 was compiled in the free open-source software Inkscape v0.92.3^75^.

## Supplementary Information

Supplementary Information.

Supplementary Figure S1.

## Data Availability

The 3D track data analysed during the current study are available in the Figshare repository: Fig. 3: https://doi.org/10.6084/m9.figshare.15082233; Fig. 4: https://doi.org/10.6084/m9.figshare.15082323; Fig. 6: https://doi.org/10.6084/m9.figshare.15082128.
