# Supplementary material for: First tracks of newborn straight-tusked elephants (Palaeoloxodon antiquus)
Source: Sci Rep. 2021 Sep 16;11:17311. doi: 10.1038/s41598-021-96754-1 (PMC8445925; doi:10.1038/s41598-021-96754-1)
Supplement: Supplementary file 1 — Supplementary Information. [file 41598_2021_96754_MOESM1_ESM.docx]

Supplementary Material

Table S1 – Proboscidean tracks and attributed trackmakers described in the fossil record in comparison to the MTS morphotype

| Description & dimensions (cm) | Ichnotaxa | Preservation | Stratigraphy & Locality | Attributed trackmaker | Reference |
| --- | --- | --- | --- | --- | --- |
| Tracks sub-circular in outline with three or more short and wide digit impressions in the *pes* with circular outline, usually visible; 11-62 | *Proboscipeda panfamilia* McNeil *et al*. 2007 | True tracks | Late Pleistocene  MIS 2  Alberta, Canada | *Mammuthus primigenius* (Blumembach 1799) | [1], [2] |
| Tracks are oval (*pes*) to circular (*manus*) 30-65; *pes* overstepping *manus*; 3 to 5 short, blunt, anteriorly-directed digit impressions | *Proboscipeda panfamilia* McNeil *et al*. 2007 | True tracks, profile | Late Pleistocene MIS 2 to 3  New Mexico, Oregon, USA | *Mammuthus columbi* (Falconer 1857) | [3], [4], [5], [6] |
| Large subcircular to subovoidal footprints, 23-27x23-30, moderate heteropody with *manus* wider than long and slightly larger than *pes* (length/width ratio ≥1). Three-to-five-digit impressions either in *manus* and *pes*, tangentially arranged along the anterior margin of the track, appearing as a series of slight undulations and/or differentiated hoofprints; broad and rounded metapodial cushion, conforming a wide flattened, but rough and cracked surface | *Proboscipeda australis* (Aramayo & Manera de Bianco 1987) | True tracks | Late Pleistocene  Buenos Aires, Argentina | *Stegomastodon platensis* (Ameghino 1888) | [7], [8] |
| Oval tracks, *pes* overprinting *manus*, with up to 4-digit impressions well marked | cf. *Proboscipeda* | True tracks | Late Pleistocene  Nagano, Japan | *Palaeoloxodon naumanni* (Makyiama 1924) | [9] |
| Large, circular to near-circular tracks; tracks are typically longer than they are wide, but some have a width equal to length; diameters ranging from 19–63;  displacement rims and radial fractures were  common and often encompassed the track; toe impressions and overprinting were rarely  observed. | *Proboscipeda* isp. | True tracks | Late Pleistocene MIS 5  Alathar, Saudi Arabia | *? Palaeoloxodon recki* (Dietrich, 1894) | [10] |
| *Manus-pes* couples with overlapping *pes*, ovoidal shape, 20x25 | *Proboscipeda panfamilia*  McNeil *et al.* 2007 | True tracks, profile, casts | Middle-Late Pleistocene MIS 6 to 5  Sardinia, Italy | *Mammuthus lamarmorai* (Major 1883) | [11] |
| Rounded-to-elliptical tracks, with an axial length range from 9.6 to 54,5 (true tracks) and to 80 (undertacks), were found mostly isolated and as paired tracks; *pes* are elliptical to sub-rounded and *manus* are circular; anteriorly directed, wide, short and blunt toe imprints, up to five; sharp edges of the toe impressions indicate the presence of nails; narrow stride | *Proboscipeda panfamilia*  McNeil *et al*. 2007 | True tracks, profile, casts | Middle-Late Pleistocene MIS 6 to MIS 4  SW Portugal, Gibraltar, SW Spain | *Palaeoloxodon antiquus* (Falconer & Cautley 1847) | [12], [13], [14], [15], [16], present work |
| Rounded to oval tracks in such case width larger than length; four toe imprints at the anterior end of the *manus* impression with sharp edges; deepest zone corresponds to the front part; 25x33 | *Proboscipeda panfamilia*  McNeil *et al*. 2007 | True tracks | Middle Pleistocene MIS 10  Roccamonfina, Italy | *?Palaeoloxodon antiquus (Falconer & Cautley 1847)* | [17] |
| Almost round *manus* 20-33 to 70 (undertacks); narrower oval *pes* 38x27; narrow stride; *pes* overprinting *manus*; toe prints generally not preserved | - | True tracks, profile, casts | Middle to Late Pleistocene MIS 11 to 3  Still Bay, Cape south coast, South Africa | *Loxodonta africana*  (Blumembach 1797)  *? Loxodonta atlantica* (Pomel, 1879) | [18], [19], [20], [21], [22], [23], [24], [25] |
| 30 in diameter, sub-circular, at least 3-digit impressions preserved, strides 200-220 | cf. *Proboscipeda* | True tracks | Early Pleistocene  Kinki, Nagano, Japan | *Stegomastodon aurorae* (Matsumoto 1918) | [9] |
| Oval footprint shape and well-defined digits | *Proboscipeda enigmatica* Panin & Avram 1962 |  | Early Pliocene  California, USA | *Gomphotherium* sp. | [26], [27] |
| Deep circular depressions with only slight toe outlines preserved 42-52 diameter; *pes* overstepping *manus* | *Proboscipeda* isp. | True tracks | Late Middle-Late Miocene  Arizona, California, USA | - | [28], [29] |
| Circular tracks without digit impression, wide trackways, stride length of 300, *pes* overprinting *manus* | *-* | True tracks | Late Miocene  Mleisa 1, United Arab Emirates | *Stegotetrabelodon syrticus* Petrocchi 1941 | [30], [31] |
| Track with 5 digital circular impressions, 20x18; deepest impression in the central pad | *Proboscipeda* isp. | True tracks | Late Miocene  Murcia, Spain | Proboscidean | [32] |
| Tracks are oval (longer in length than width), 14-17, with  pockmarked texture, toe impressions generally absent, though  rare tracks may show very small toe impressions on the anterior  or lateral side. | *Proboscipeda enigmatica* Panin & Avram 1962 | True tracks | Middle Miocene  Vrancea, Romania | Deinothere | [33] |
| Circular to  oval *manus* and *pes* impressions; some  of the footprints are overstepped,  14.9×11.5; three digits imprints are visible in the two  pes-imprints as crescent shapes. | *Proboscipeda enigmatica* Panin & Avram 1962 | True tracks | Late Eocene  Iran | Moeritheriidae, Barytheriidae | [34] |

References

1. McNeil, P., Hills, L. V., Kooyman, B. & Tolman, S. M. Mammoth tracks indicate a declining Late Pleistocene population in southwestern Alberta, Canada. *Quat. Sc. Rev.* 24, 1253-1259 (2005).

2. McNeil, P., Hills, L. V., Tolman, S. M. & Kooyman, B. Significance of latest Pleistocene tracks, trackways and trample grounds from southwestern Alberta, Canada. *Can. Bull. Nat. H. Mus. Nat. Hist. Sci.* 42, 209-224 (2007).

3. Lucas, S.G. *et al*. Mammoth footprints from the Upper Pleistocene of the Tularosa basin, Doñana county, New Mexico. In *Cenozoic vertebrate tracks and traces* (eds. Lucas, S.G., Spielmann, Lockley, M.G.) 149-154 (New Mexico Mus. Nat. Hist. Sc. 42, 2007).

4. Retallack, G. J. *et al*. Late Pleistocene mammoth trackway from Fossil Lake, Oregon. *Palaeogeogr. Palaeoclimatol. Palaeoecol.* 496, 192-204 (2018).

5. Bennett, M. R. *et al*. Soft-sediment deformation below mammoth tracks at White Sands National Monument (New Mexico) with implications for biomechanical inferences from tracks. *Palaeogeogr. Palaeoclimatol. Palaeoecol*. 527, 25-38 (2019).

6. Urban, T. M. *et al*. 3-D radar imaging unlocks the untapped behavioral and biomechanical archive of Pleistocene ghost tracks. *Sci. Rep*. 9, 16470; 10.1038/s41598-019-52996-8 (2019).

7. Aramayo, S. A., Manera de Bianco, T., Bastianelli, N. V. & Melchor, R. N. Pehuen Co: updated taxonomic review of a late Pleistocene ichnological site in Argentina. *Palaeogeogr. Palaeoclimatol. Palaeoecol*. 439, 144-165 (2015).

8. Oliva, C. & Arregui, M. Mammalian Ichnopathology: a case study of Holartic ungulates (Gomphotheriidae, Equidae, Camelidae) of the Late Pleistocene of South America. Ichnotaxonomic implications. *Bol. Soc. Geol. Mex*. 70(2), 417-447 (2018).

9. Matsukawa, M. & Shibata, K. Review of Japanese Cenozoic (Miocene-Modern) vertebrate tracks. *Ichnos* 22, 261-290 (2015).

10. Stewart, M. *et al*. Human footprints provide snapshot of last interglacial ecology in the Arabian interior. Sci. Adv. 2020; 6 : eaba8940 (2020).

11. Pillola, G.L. & Zoboli, D. Dwarf mammoth footprints from the Pleistocene of Gonnesa (southwestern Sardinia, Italy). *Boll. Soc. Paleont. Ital*. 56, 57-64 (2017).

12. Neto de Carvalho, C. Vertebrate tracksites from the Mid-Late Pleistocene eolianites of Portugal: the first record of elephant tracks in Europe. *Geol. Quart*. 53(4), 407-414 (2009).

13. Neto de Carvalho, C., Figueiredo, S. & Belo, J. Vertebrate tracks and trackways from the Pleistocene eolianites of SW Portugal. *Comun. Geol*. 103 (Esp. I), 101-116 (2016).

14. Muñiz, F. *et al*. Following the last Neanderthals: Mammal tracks in Late Pleistocene coastal dunes of Gibraltar (S Iberian Peninsula). *Quat. Sc. Rev*. 217, 297-309 (2019).

15. Neto de Carvalho, C. *et al*. Tracking the last elephants in Europe during the Würm Pleniglacial: the importance of the Late Pleistocene aeolianite record in SW Iberia. *Ichnos* 27(3), 352-360 (2020a).

16. Neto de Carvalho, C. *et al*. First vertebrate tracks and palaeoenvironment in a MIS-5 context in the Doñana National Park (Huelva, SW Spain). *Quat. Sc. Rev*., 243, 10.1016/j.quascirev.2020.106508 (2020b).

17. Palombo, M. R., Panarello, A. & Mietto, P. Did elephants meet humans along the Devil’s path? A preliminary report. *Alp. Med. Quat*. 31, 83-87 (2018).

18. Roberts, D. L., Bateman, M. D., Murray-Wallace, C. V., Carr, A. S. & Holmes, P. J. Last interglacial fossil elephant trackway dated by OSL/AAR in coastal aeolianites, Still Bay, South Africa. *Palaeogeogr. Palaeoclimatol. Palaeoecol*. 257, 261-279 (2008).

19. Helm, C.W. *et al*. A new Pleistocene hominin tracksite from the Cape south coast, South *Africa. Sci. Rep*. 8, 3772 (2018a).

20. Helm, C.W. *et al*. Late Pleistocene vertebrate trace fossils in the Goukamma Nature Reserve, Cape south coast, South Africa. *Paleont. Afr*. 52, 89-101 (2018b).

21. Helm, C.W. *et al*. The Pleistocene fauna of the Cape south coast revealed through ichnology at two localities. *S. Afr. J. Sc*. 115, 5135 (2019a).

22. Helm, C.W. *et al*. Pleistocene vertebrate trace fossils of Robberg Nature Reserve. *Paleont. Afr*. 54, 36-47 (2019b).

23. Helm, C.W. et al. Pleistocene vertebrate tracksites on the Cape south coast of South Africa and their potential paleoecological implications. *Quat. Sc. Rev.* 235, 105957 (2020).

24. Helm, C.W., Cawthra, H.C., De Vynck, J.C., Dixon, M. & Stear, W. Elephant tracks: a biogenic cause of potholes in Pleistocene South African coastal rocks. *J. Coast. Res*. 37, 59-74 (2021a).

25. Helm, C.W. *et al*. Morphology of Pleistocene elephant tracks on South Africa’s cape south coast and probable elephant trunk-drag impressions. *Quat. Res.* 1-15, doi.10.1017/qua.2021.32 (2021b).

26. Remeika, P. The Fish Creek Canyon ichnofauna: a Pliocene (Blancan) vertebrate footprint assemblage from Anza-Borrego Desert State Park, California. In *Proceedings of the 6th Fossil Resource Conference* (eds. Santucci, V.L. and McClelland, L.) 55-75 (Geological Resources Division Technical Report NPS/NRGRD/GRDTR-01/01, National Park Service D-2228, 2001).

27. Remeika, P. Fossil footprints of Anza-Borrego in Fossil Treasures of the Anza-Borrego Desert: The Last Seven Million Years. In *Fossil treasures of the Anza-Borrego Desert* (eds. Jefferson, G.T. and L. Lindsay), 311-327 (California State Parks, The Anza-Borrego Foundation and Institute, and Sunbelt Publications, San Diego, California, 2006).

28. Brady, L. F. & Seff, P. Elephant Hill. Plateau 31, 80-82 (1959).

29. Scrivner, P.J. & Bottjer, D.J. Neogene avian and mammalian tracks from Death Valley National Monument, California: Their context, classification and preservation. *Palaeogeogr. Palaeoclimatol. Palaeoecol*. 57(2-4), 285-331 (1986).

30. Higgs, W., Kirkham, A. Evans, G. & Hull, D. A Late Miocene Proboscidean trackway from western Abu Dhabi. *Tribulus* 13(2), 3-8 (2003).

31. Bibi, F. *et al*. Early evidence for complex social structure in Proboscidea from a late Miocene trackway site in the United Arab Emirates. *Biol. Lett*. 8, 670-673 (2012).

32. Pérez-Lorente, F., Rodríguez, T., Mancheño, M.A., Aerrano, F. & Romero, M. Pisadas fósiles de mamíferos en el Mioceno superior de La Hoya de la Sima (Jumilla, Murcía, España). *Mem. de Arqueol.* 12, 15-28 (1999).

33. Panin, N. & Avram, E. Noe urne de vertebrate in Miocenul Subcarpatilor rominesti. *St. Cerc. Geol.* 7, 455-484 (1962).

34. Abbassi, N., Alinasiri, S. & Lucas, S. G. New localities of Late Eocene vertebrate footprints from the Tarom Mountains, Northwestern Iran. *Hist. Biol.* 29(7), 987-1006 (2017).
