# Supplementary material for: First tracks of newborn straight-tusked elephants (Palaeoloxodon antiquus)
Source: Sci Rep. 2021 Sep 16;11:17311. doi: 10.1038/s41598-021-96754-1 (PMC8445925; doi:10.1038/s41598-021-96754-1)
Supplement: Supplementary file 2 — Supplementary Figure S1. [file 41598_2021_96754_MOESM2_ESM.docx]

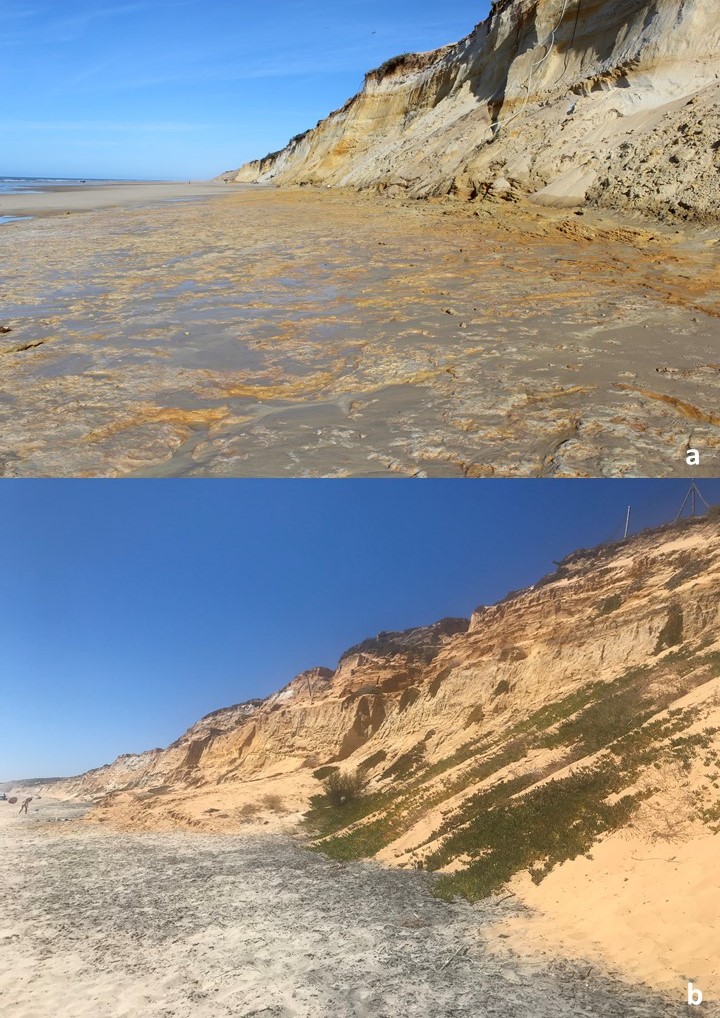


Fig. S1 – MTS is almost always covered by a thick blanket of beach sand and remobilized sand resulting from the fast erosion of the cliff front. The photos taken in October 2020 (a) and July 2021 (b) show that the wide exposure of the MTS in June 2020 was an extraordinary but ephemeral event (photos of J.M. Galán and C.N. Carvalho).
